# Correlation of the HPV 16 Genotype Persistence in Women Undergoing LEEP for CIN3 with the Risk of CIN2+ Relapses in the First 18 Months of Follow-Up: A Multicenter Retrospective Study

**DOI:** 10.3390/diagnostics14050509

**Published:** 2024-02-28

**Authors:** Maria Teresa Bruno, Gaetano Valenti, Zaira Ruggeri, Giosuè Giordano Incognito, Paola Coretti, Giuseppe Dario Montana, Marco Marzio Panella, Liliana Mereu

**Affiliations:** 1 Gynecology and Obstetrics Unit, Department of General Surgery and Medical-Surgical Specialty, Rodolico University Hospital, University of Catania, 95123 Catania, Italy; 2 Multidisciplinary Research Center in Papillomavirus Pathology, Chirmed, University of Catania, 95123 Catania, Italy; 3 Humanitas, Gynaecologic Oncology Unit, 95125 Catania, Italy; 4 Cervical Cancer Screening Unit, Level II, ASP Messina, 98123 Messina, Italy

**Keywords:** HPV persistence, recurrence, LEEP, CIN2+, relapse, follow-up

## Abstract

Objective: Specific hr-HPV genotypes have different natural histories and different oncogenic capacity. This study aimed to investigate the risk of CIN2+ recurrence of the individual genotypes and evaluate how the duration of HPV persistence influences the risk of developing recurrent 16 cervical dysplasia of high grade (CIN2+). Methods: Data from patients with persistent HPV infection after primary conization were retrospectively extracted. Kaplan-Meier proportional hazards models were used to evaluate associations between the duration of HPV persistence and the risk of developing recurrent CIN2+. Kruskal-Wallis testing with Dunn’s multiple comparison test was used to test whether there was a statistically significant difference in the time to development of tumor recurrences between different genotypes. Results: Overall, 333 patients met the inclusion criteria. In 285 cases the HPV infection was persistent, in 48 cases (18%) it was transient, i.e., different genotypes after LEEP. Overall were diagnosed 39 relapses (13.7%), 79.5% (31/39 cases) were due to genotype 16, 20.5% (8/39) were linked to the other genotypes. Persistence of genotype 16 showed a 7-fold increased risk of developing a CIN2+ relapse, OR = 7.08 (95%CI: 3.12–16.08). Furthermore, the majority of relapses (38/39) occurred within 24 months of persistence with a cut-off represented by 18 months (*p* = 0.001) in which the relapse rate is maximum and the most frequently found genotype was the 16th with 31 (79.5%) cases of recurrence. Kruskal-Wallis test with Dunn’s multiple comparisons has shown statistically significant difference in the time of development of CIN2 relapses among HPV16 and other genotypes. (*p* < 0.05). Kaplan-meier analysis has shown statistically significant difference between the time to CIN2+ relapse onset in patients with HPV 16 infection and patients with other hrHPV genotypes. (*p* < 0.05) Conclusions: the study results suggest that persistent HPV infection after LEEP with the same HR genotype present before surgery represents one of the most important predictive factors of the risk of CIN2+ recurrence. The persistence of HPV16 for the first 18 months strongly correlates with the risk of developing a CIN2+ recurrence.

## 1. Introduction

Papillomavirus infection is the most common sexually transmitted infection in the world with prevalence rates varying between different geographical regions depending on their level of development and the characteristics of the population studied. It is estimated that 50–80% of sexually active men and women will contract a genital HPV infection in their lifetime [1]. Its affinity for cervical squamous cells has made high-risk genotypes (hr HPV) closely related to cervical cancer (SCC) [2,3,4]. Genotypes 16 and 18 are the most endowed with oncogenic capacity, HPV 16 alone is responsible for over 50% of cervical carcinomas and both cause 70% of cervical carcinomas. HPV infection is also associated with anal, head and neck cancers, and some cancers of the vulva, penis, and anus [5,6,7]. HPV 6 and 11 are related to recurrent respiratory papillomatosis in children, as well as approximately 90% of genital warts [8]. Studies conducted in recent decades have shown that it is capable of infecting and replicating in syncytiotrophoblast cells, causing adverse events in pregnancy (miscarriage, preeclampsia and preterm birth) [9,10,11,12]. In Italy the prevalence of HPV infection in women with a normal Pap test is 7.8%, while genotype 16 in women with cytological anomalies has a prevalence between 15.6% of the CIN1 lesion up to 61.2% of carcinoma invasive [13].

### 1.1. Role of Persistent HPV-hr Infection after LEEP

Its incidence is greater in younger populations, adolescents and young women making their sexual debut. In 80% of cases, within 18–24 months, the virus is eliminated. An undetectable HPV infection could be a period of viral latency, where HPV levels are below the detectable threshold of current HPV DNA tests, rather than representing viral clearance. HPV infection is defined as persistent if HPV was detected in two consecutive follow-up visits 6 months apart. High-risk genotypes, especially HPV16, adopt a strategy to evade the defense mechanisms of the host, which becomes indifferent to the presence of the virus. Persistent papillomavirus infection is a risk factor for the development of cervical intraepithelial neoplasia (CIN) and cervical cancer [14]. The persistence of high-risk genotypes identifies women at risk of preneoplastic lesions which, if not diagnosed and treated in time, can progress to cervical cancer [15,16]. The knowledge of the natural history of cervical cancer and papillomavirus acquired in recent decades, the tests implemented for its diagnosis, together with screening and HPV vaccination give us hope of defeating cervical cancer by 2030 [17,18,19,20]. CIN3/HSIL, the true precursor of cervical cancer, must be treated with excisional methods that can be performed with a cold blade or by LEEP. Although this treatment is generally sufficient and leads to complete recovery, the literature reports an average of 10% residual or recurrent disease [21].

### 1.2. Risk Factor for HPV-hr Previously Reported after LEEP

Postoperative persistence of HPV infection is defined as the presence of the same HPV genotype before LEEP and at the first follow-up visit after surgery. Increasing evidence has shown that persistence of HPV infection after cervical excision is closely related to recurrence and even disease progression and may be an independent factor of recurrence [22,23]. It is therefore essential that all women undergoing LEEP for CIN3 undergo HPV testing to study their viral status. This supports current American Society for Colposcopy guidelines for the use of post-treatment HPV testing as a follow-up option to identify women at high risk of cervical cancer recurrence after treatment [21]. The positive HPV DNA test highlights the presence of hr-HPV which can result from the persistence of the same genotype or from reinfection with a new type of hr-HPV, especially in young women. hr-HPV genotyping allows us to differentiate persistent from transient infection.

### 1.3. The Shortage of Previous Study and Aim of This Study

Considering that specific hr-HPV genotypes have different natural histories and different oncogenic capacity, the objective of our study is to evaluate the risk of CIN2+ relapses of single persistent genotypes in a sample of women undergoing LEEP for CIN3. Furthermore, since no data are available on the correlation between the duration of HPV persistence and the risk of CIN2+ recurrence, the study aim to identify the follow-up period most at risk of recurrence.

## 2. Materials and Methods

### 2.1. Study and Design Population

#### 2.1.1. Reference Population

A multicenter retrospective study was conducted; data from 956 women undergoing cervical conization (LEEP) for CIN from January 2012 to December 2018 were collected in a dedicated database. Inclusion criteria were: women who underwent LEEP for CIN3, who had an HPV test and HPV genotyping before and after LEEP and who completed a 4-year follow-up.

Women who underwent LEEP for histology other than CIN3, who had no follow-up data, who had a history of cervical cancer or history of HIV or other conditions affecting the immune response were excluded.

A ThinPrep PreservCyt cervical specimen (Hologic, Inc, Bedford, MA, USA) was collected in all patients at baseline and post-treatment visits for HPV DNA testing and HPV genotyping.

All women underwent loop electrosurgical excision procedure (LEEP) treatment.

Residual disease is the diagnosis of CIN2+ at the first post-conization evaluation.

Recurrence or recurrences occur when the CIN2+ lesion is diagnosed after a negative test.

Identification of the same HPV genotype before and after LEEP was considered HPV-specific persistence.

Incident or transient HPV infections are defined as the detection of a new HPV genotype after treatment for CIN3.

The ethics committee of the University Hospital (Catania 2) waived the obligation of ethical approval and informed consent because the study used previously archived data, according to current legislation (20 March 2008) (AIFA).

According to Italian law, patient consent was not mandatory in a retrospective study [24].

#### 2.1.2. Follow-Up Procedure

The follow-up protocol included repeating the Pap test/HPV test followed by colposcopy every 6 months for two years and then every year; if three subsequent co-tests were negative, the frequency was every three years.

Pap smears were interpreted with the Bethesda system and histological diagnoses of the excised specimens were based on the World Health Organization classification.

All included centers used the same molecular technique for HPV DNA detection and genotyping. The guidelines proposed by the Italian Society of Colposcopy and Cervico-Vaginal Pathology were followed for the diagnosis, treatment and follow-up of the study population.

### 2.2. LEEP Technique

LEEP was performed with colposcopy guidance under local anesthesia in the clinic by experienced staff; Loops 20 mm wide and 12, 15 or 20 mm deep were used depending on the characteristics of the lesion and the conformity of the cervix. Resection margins were maintained 2–3 mm beyond the lesion, and completeness of lesion removal was colposcopically verified. Histological examination of the cervical cone established the definitive histological diagnosis and assessed the involvement of the cone margins. Cone margins were reported as positive if the distance between CIN2+ and the resection surface was <1 mm.

### 2.3. HPV Testing and Genotyping

After cytological sampling for HPV DNA, samples were sent to the laboratory for DNA extraction [16] and genotyping of viral DNA by genetic amplification followed by hybridization with genotype-specific probes capable of identifying most HPV genotypes of the genital region. Automated DNA extraction was carried out with a 1 mL sample using the NucliSENS EASYMAG system (bioMérieux SA, Marcy l’Etoile, France) following the manufacturer’s HPV 1.1 protocol, with a 55 μL final elution volume.

Amplification of HPV DNA was accomplished by HPV-HS Bio (AB Analiticas.r.l, Padova, Italy) nested polymerase chain reaction (PCR) for the detection of HPV DNA sequences within the L1 open reading frame (ORF), according to the manufacturer’s recommendations. To verify the efficiency of the DNA extraction, the housekeeping gene thiosulphate-sulphur transferase (TST) was also amplified. Samples negative for TST were considered inadequate and a new sample was requested.

For the first-amplification step, carried out with 10 µL of eluate, a combination of degenerate primers was used to amplify a 449–458 bp sequence within the L1 ORF of the HPV genome. The second amplification was carried out on 1 µL of the first amplification product, using biotinylated primers to amplify a 139–145 bp sequence. To verify the efficiency of the DNA extraction, 10 μL of eluate were used to amplify a 202 bp fragment of the TST gene using specific primers. Negative (water) and positive controls (plasmid clones containing HPV54) were included for each PCR run to check for accuracy and possible contamination. To confirm amplification, PCR products were submitted to electrophoresis in 3% agarose gel, and the positive ones were used for the hybridisation step. Samples negative for TST were considered inadequate and extracted again from the second tube. For all the positive samples at a reverse line blot hybridisation assay, HPV typing was carried out with specific probes for the most frequent HPV types (HPV type, AB Analitica s.r.l., Padova, Italy). HPV types allows the identification of 11 LR genotypes (6, 11, 40, 42, 43, 44, 54, 61, 70, 72, 81) and 18 HR genotypes (16, 18, 26, 31, 33, 35, 39, 45, 51, 52, 53, 56, 58, 59, 66, 68, 73, 82). Samples that were positive by nested PCR but negative in reverse line blot for any of these types were considered as undetermined HPV.

#### Statistical Analysis

The Shapiro-Wilk test was used to check whether the data adhered to the normal distribution.

Odds ratios were calculated to identify whether the HPV genotype had a higher risk of developing tumor recurrence, reinfection due to a different genotype, or unachieved viral clearance.

Kaplan-Meier analysis was used to identify a possible statistically significant difference between the time to onset of recurrence in patients with HPV 16 infection and in patients with other hrHPV genotypes.

Kruskal-Wallis testing with Dunn’s multiple comparison test was used to test whether there was a statistically significant difference in the time to development of CIN2 recurrences between different genotypes.

Values of *p* < 0.05 were considered statistically significant. Statistical analysis was performed using JASP software (version 0.18.10) and GraphPad Prism version 10.0.0 for Windows, GraphPad Software, Boston, MA USA, www.graphpad.com (accessed on 13 December 2023).

## 3. Results

Of the 956 patients who underwent LEEP, only 859 met the requirements: 47 cases with a histological diagnosis other than CIN3 and 52 women who required hysterectomy were excluded.

After primary LEEP, 333 (38.8%) women were HPV positive; in 285 cases HPV infection was persistent, in 48 cases (18%) the infection was transient, i.e., different genotypes after LEEP.

**Figure 1:**
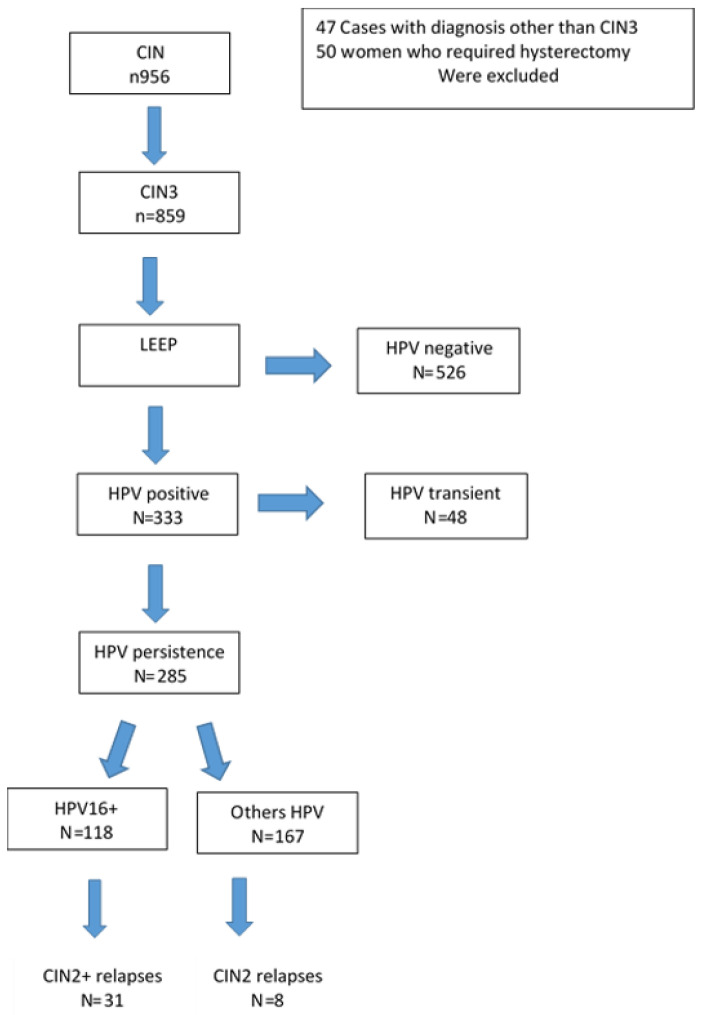
Flow-Chart of study population.

Of the 285 (33.2%) women with persistent infection, 118 (41.4%) had genotype 16 and 167 (58.6%) were positive for genotypes 18, 31, 33, 45, 51, 52, 56 (Figure 1).

Table 1 shows the characteristics of the study population.

Table 2 shows the percentage of the genotypes detected in the study population: 41.4% (118/285) of the sample consisted of women positive for genotype 16, 18.2% (52 cases) for women positive for genotype 31, 32 cases (11.2%) had genotype 18, 27 (9.5%) women were positive for 33, followed by HPV 45 with 20 cases (7%), HPV 51 with 12 cases (4.2%), HPV 52 with 15 cases (5.3%), HPV 56 with six cases (2.1%), and HPV 35 with 3 cases (1.0%). The overall viral persistence rate after primary LEEP was 33.2% (285/859 cases) and 13.7% (39/285 cases) relapse.

At 12 months after LEEP the overall persistence was 56.1% (160/285 cases), at 18 months it was 31.6% with 90 cases of persistence, at 24 months the persistence rate was 20.7% (59 cases), at 36 months it was 11.2% (32 cases), at 48 months there were only 12 (4.2%) persistent HPV cases.

Considering genotype-specific persistence, the HPV 16 group had a persistence rate of 80.5%, 46.6%, 33.9%, 20.3%, 8.5% at 12, 18, 24, respectively. 36 and 48 months. Genotype 31 had a persistence rate of 48.1%, 34.6%, 19.2%, 7.7%, 0% at 12, 24, 36, and 48 months, respectively.

Overall, the study revealed 39 recurrences (13.7%), 79.5% (31/39 cases) due to genotype 16, 20.5% (8/39) were related to the other genotypes. Persistence of genotype 16 showed a 7-fold increased risk of developing CIN2+ recurrence, OR = 7.08 (95%CI: 3.12–16.08). Odds ratios calculated regarding the other genotypes and the risk for the development of CIN2+ relapses has not shown statistically significant results. (*p* > 0.05) Table 3.

As regards the relationship between the duration of persistence and the appearance of relapse, the highest relapse rate occurred at 18 months with 22 (56.4%) cases, while the most correlated genotype was HPV16 with 31 cases, followed by genotype 18 with 3 cases, from HPV 31 with two cases and from HPV 45 and HPV 52 with one case each. Therefore, the majority of relapses (38/39) occurred within 24 months of persistence with a cut-off at 18 months (22 relapses) and the most frequently found genotype was 16 with 31 (79.5%) cases of relapse.

**Figure 2:**
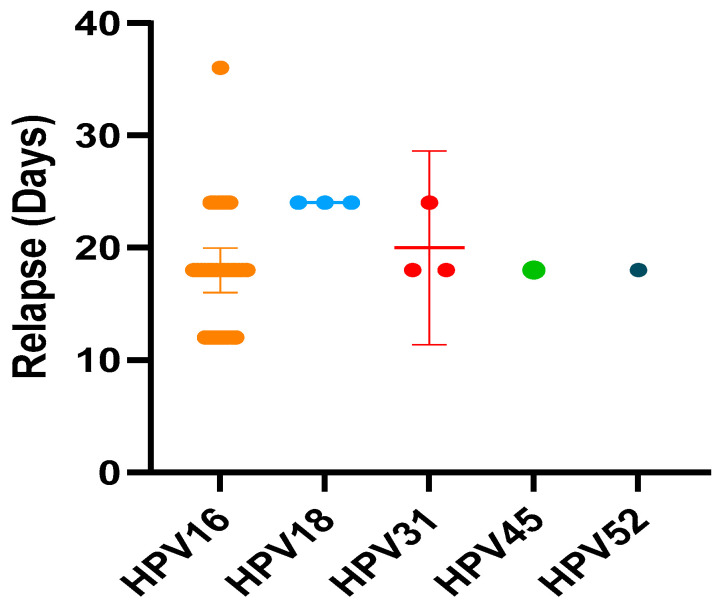
The time of development of CIN2 relapses among HPV16 and others genotypes. (Kruskal-Wallis test with Dunn’s multiple comparisons).

Kruskal-Wallis test with Dunn’s multiple comparisons has shown statistically significant difference in the time of development of CIN2 relapses among HPV16 and others genotypes (*p* < 0.05) (Figure 2).

**Figure 3:**
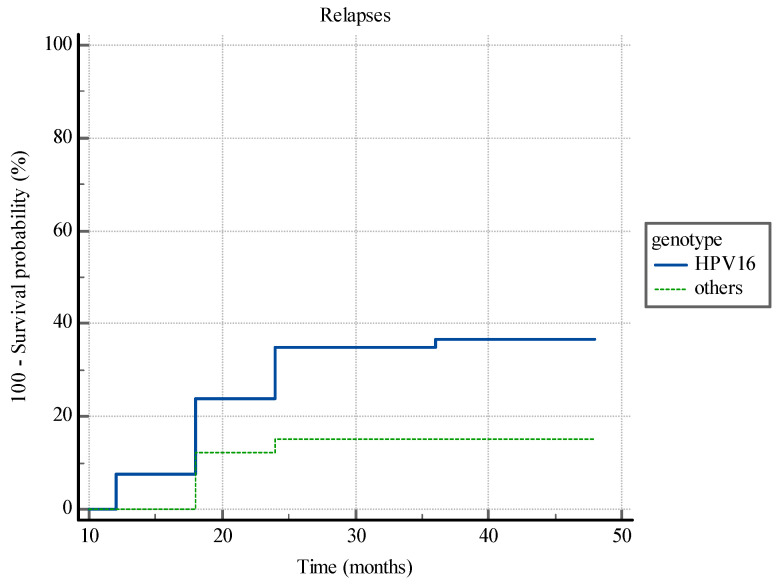
Kaplan-Meier analysis. The time to CIN2+ relapse insurgence in patients with HPV 16 infection and patients with other hrHPV genotypes.

Kaplan-Meier analysis has shown statistically significant difference between the time to CIN2+ relapse insurgence in patients with HPV 16 infection and patients with other hrHPV genotypes (*p* < 0.0013) (Figure 3).

The women were followed for 4 years and 12/223 women (5.4%) were still positive for the same type of HPV detected before treatment. The 39 women with relapse underwent second LEEP or hysterectomy.

No recurrence was observed among patients infected with newly detected types of hrHPV after LEEP.

## 4. Discussion

Literature data supports that viral persistence after conization is a risk factor for the development of relapses. In this study, the existence of a correlation between specific HPV genotypes and the onset of relapses was investigated. The results of present study confirm that persistent HPV infection after treatment is a significant independent predictor for the development of CIN2+ recurrence. In particular, the risk of CIN2+ recurrence was higher in patients with a persistent HPV 16 infection than in other genotypes. The results of this study add to the data from the literature on the usefulness of HPV typing in monitoring women during follow-up [25,26].

Furthermore, there is no data on the existing correlation between the duration of HPV persistence and the onset of relapses. The study shows that there is a correlation between the length of persistence and relapses.

The present study had an overall viral persistence at 6 months of 33.2%; other authors have reported lower 6-month HPV persistence rates, ranging from 14.3% to 21.5% [27]. The variability between studies is due to the lack of homogeneity of the data included in the various studies; some authors have considered CIN of different degrees and the method of detecting the virus changes between studies [28]. The higher persistence rate is related to the selection of only CIN3 patients and the fact that the majority of patients in the present study were positive for the five most oncogenic genotypes (16, 18, 31, 33, 45). In addition, it is known that the percentage of women who show persistence of HPV after treatment varies depending on the method used. A follow-up study after CO2 laser conization found 100% clearance of the initial HPV genotype; 9% of patients treated with knife conization had persistent HPV 12 months after surgery [29,30]. Other studies have found HPV persistence of 20.4% and 35.1% after LEEP, the latter finding being very similar to ours [26,31].

Median estimates of global HPV persistence tended to decrease as follow-up time after LEEP increased. At the end of the four years of follow-up, the sample of women with persistent HPV after LEEP showed 13.7% progression, 82% regression and 4.2% persistence.

The objective of the type-specific HPV test is to distinguish, after LEEP, the persistence of the same genotype involved in the CIN2+ lesion. Given that up to one third of treated women are still HPV positive at follow-up, it is necessary to distinguish whether this persistence is related to the same original genotype or belongs to a new infection. Whereas younger women may have a high rate of transient infection after treatment.

In the study, more extensive genotyping and longer follow-up were used than in most studies [32,33], which allowed the viral status to be adequately monitored and investigate whether an infection during follow-up was of the newly acquired type or whether the genotype was the same as before treatment. In the literature, estimates of the persistence of the single genotype associated with the probability of developing a CIN2+ relapses are very limited. Most studies do not distinguish between recurrent or residual cervical disease, and most of them do not differentiate between persistent and newly acquired lesions. In the present study, the postoperative HPV genotypes were the same as the preoperative ones, and the authors reported the type-specific persistence of HPV 16, 18 and 31, the most common genotypes in the series considered. This allowed to study the ability of individual genotypes to recur and to identify which time interval of follow-up was most correlated with the risk of relapses.

The results of present study show that persistent HPV 16 infection was significantly associated with CIN2+ relapses (*p* < 0.001).

Persistence of genotype 16 showed a 7-fold increased probability of developing CIN2+ relapses, OR = 7.08 (95% CI 3.12–16.08). HPV 31 showed no significant correlation with CIN2+ relapses (*p* = 0.07), the same goes for genotype 18 (*p* = 0.45) probably due to the different genotypes clearance rate of the genotypes after treatment. Elimination of HPV usually occurs within three months of surgery, while HPV16 tends to disappear slowly and less than other oncogenic types [34]. The results confirm the clinical evidence of the ALTS study and others, in which the persistence of HPV16 associated with disease relapse was more frequently detected [26,34,35,36]. Therefore, women with persistent HPV 16 infection may benefit from more intensive postsurgical follow-up, including immediate colposcopy after treatment and closer testing, in order to identify any relapses early.

The results of the study shows that there is a correlation between the length of persistence and relapses. In the first 18 months after LEEP there is a significant risk of relapse, *p* = 0.001; prolonged persistence beyond 18 months was not significant (*p* = 0.63) and did not increase the risk of developing a recurrence.

Women positive for HPV16 at 6 months after LEEP have a 7-fold greater risk of developing recurrence within 18 months, compared to those positive for HPV18 or other hr genotypes. In the following months the risk of relapse decreases significantly until it becomes non-significant. Therefore, patients with HPV 16 should be carefully monitored after LEEP. On the contrary, the post-therapy identification of an HPV genotype different from that present before LEEP could allow a reduction in the intensity of follow-up.

The data of the research support the usefulness of hr HPV genotyping before and after LEEP to manage patients with post-treatment CIN3, as also expressed by other authors and allows us a personalized treatment avoiding subjecting women infected with another genotype to treatment [27,37,38].

A limitation of the study is the retrospective design, as it generates a large amount of incomplete data and the small sample size. The small sample analyzed leads to biased estimates of the importance of different genotypes. The correlation analyze between specific genotypes and risk of recurrence have shown differences in the hierarchy of HPV types, improving HPV31 and HPV52 and downgrading HPV18 and HPV45 while we know from numerous studies that HPV18 and HPV45 have a higher oncogenic risk than to any other type, after genotype16 [9,31]. The strengths of the study are the homogeneity of the data such as the fact that only patients affected by CIN3, the most exposed to relapse after LEEP and progression of the disease, were selected. Furthermore, the use of more extensive genotyping and follow-up allowed to carefully study the viral status of the study sample and to differentiate women with the same genotype as before LEEP from those with transient infection.

## 5. Conclusions

The findings suggest that particular attention should be paid to persistent HPV infection after LEEP with the same type of hr HPV present before surgery. It is therefore important to subject women to HPV genotyping prior to LEEP. Persistence of HPV16 within 18 months is strongly correlated with the risk of developing CIN2+ recurrence. Detection of persistent HPV 16 infection after treatment should be considered as the main risk factor for the development of recurrent CIN2+. Recent studies support the usefulness of HPV vaccination after treatment, but large prospective studies are needed [38,39].

## Figures and Tables

**Table 1:** Characteristics of the study population after LEEP and during the follow-up time.

Age	38 (24–56)	
HPV Infection after LEEP		
Persistence	285 (33.2%)	
Clearance	526 (61.2%)	
Transient	48 (5.6%)	
Persistence infection for specific HPV genotypes		
HPV16	118 (41.4%)	
HPV18	31 (11.2%)	
HPV31	52 (18.2%)	
HPV33	27 (9.5%)	
HPV45	20 (7%)	
HPV51	12 (4.2%)	
HPV52	15 (5.3%)	
HPV56	56 (2.1%)	
HPV35	2 (1%)	
Global persistence for F.U time		
12 mounts	160 (56.1%)	
18 mounts	90 (31.6%)	
24 mounts	59 (20.7%)	
36 mounts	32 (11.2%)	
48 mounts	12 (4.2%)	
Global recurrence	39 (13.7%)	
Recurrence for specific genotypes		*p*
HPV16	31 (79.5%)	<0.001
HPV 18	3 (7.7%)	0.49
HPV 31	3 (7.7%)	0.45
HPV 33	0	1
HPV45	1 (2.6%)	0.24
HPV51	0	1
HPV52	1 (2.6%)	0.42
HPV56	0	1
HPV31	0	1
Recurrence for F.U time		*p*
12 mounts	9	0.001
18 mounts	22	0.001
24 mounts	7	0.6
36 mounts	1	0.4
48 mounts	0	

**Table 2:** The percentage of genotypes detected in the study population after LEEP. The correlation between specific genotypes and relapses and between duration of persistence and relapses during follow-up.

HPV Genotype	6 Months	12 Months		18 Months		24 Months		36 Months		48 Months	
	n	n	CIN2+	n	CIN2+	n	CIN2+	n	CIN2+	n	CIN2+
HPV16	118 (41.4%)	95 (80.5%)	9 (9.5%)	55 (46.6%)	15 (27.3)	40 (33.9%)	6 (15%)	24 (20.3%)	1 (4.2%)	10 (8.5)	0 (0%)
HPV31	52 (18.2%)	25 (48%)	0	18 (34.6%)	2 (11.1)	10 (19.2%)	1 (10%)	4 (7.7%)	0	0	0
HPV18	32 (11.2%)	10 (31.2%)	0	5 (13.6%)	3 (60%)	4 (12.8%)	0	3 (9.4%)	0	2 (6.2%)	0
HPV33	27 (9.5%)	14 (51.8%)	0	3 (11.1%)	0	1 (3.7%)	0	0	0	0	0
HPV45	20 (7%)	8 (40%)	0	4 (20%)	1 (25%)	1 (5%)	0	1 (5%)	0	0	0
HPV51	12 (4.2%)	4 (33.3%)	0	2 (16%)	0	1 (8.3%)	0	0	0	0	0
HPV52	15 (5.3%)	3 (20%)	0	3 (20%)	1 (33.3)	2 (14.1%)	0	0	0	0	0
HPV56	6 (2.1%)	0	0	0	0	0	0	0	0	0	0
HPV35	3 (1%)	1 (33.3%)	0	0	0	0	0	0	0	0	0
Total	285 (33.2%)	160 (56.1%)	9	90 (37.6%)	22	59 (26.5%)	7	32 (14.3%)	1	12 (5.4%)	0

**Table 3:** The risk of developing CIN2+ recurrence of HPV genotypes 16, 18 and 31 considered in the study.

Genotype	OR	CI 95%	*p*
HPV16	7.08	3.12–16.08	0.001
HPV18	0.34	0.10–1.13	0.07
HPV 31	0.65	0.19–2.25	0.49

## Data Availability

Data are available upon reasonable request.
